# DNA Methylation and gene expression patterns are widely altered in fetal growth restriction and associated with FGR development

**DOI:** 10.1080/19768354.2021.1925741

**Published:** 2021-05-17

**Authors:** Seoyeong Lee, Young Nam Kim, DoHwa Im, Su Han Cho, Jiyeon Kim, Jeong-Hyun Kim, Kwoneel Kim

**Affiliations:** a Department of Biology, Kyung Hee University, Seoul, Republic of Korea; b Department of Obstetrics and Gynecology, Busan Paik Hospital, Inje University, Busan, Republic of Korea; c Department of Life and Nanopharmaceutical Sciences, Kyung Hee University, Seoul, Republic of Korea; d Department of Medicine, University of Ulsan College of Medicine, Seoul, Republic of Korea

**Keywords:** Fetal growth restriction, DNA methylation, INS, MEG3, and ZFP36L2

## Abstract

Fetal growth restriction (FGR) is the failure of the fetus toachieve its genetically determined growth potential, which increasesrisks for a variety of genetic diseases, such as type 2 diabetes mellitus, coronary artery disease, and stroke, during the lifetime. The dysregulation of DNA methylationis known to interact with environmental fluctuations, affect gene expressions comprehensively, and be fatal to fetus development in specific cases. Therefore, we set out to find out epigenetic and transcriptomic alterations associated with FGR development. We found a set of differentially expressed genes associated with differentially methylated regions in placentae and cord blood samples. Using dimensional reduction analysis, the expression and methylation variables of the epigenetically altered genes classified the FGR samples from the controls. These genes were also enriched in the biological pathways such as metabolism and developmental processes related to FGR. Furthermore, three genes of INS, MEG3, and ZFP36L2 are implicated in epigenetic imprinting, which has been associated with FGR. These results strongly suggest that DNA methylation is highly dysregulated during FGR development, and abnormal DNA methylation patterns are likely to alter gene expression.

## Introduction

Fetal growth restriction (FGR) is the failure of the fetus to achieve its genetically determined growth potential and is defined as estimated fetal weight less than the 10th percentile (Minior and Divon 1998; American College of Obstetricians 2013). Small-for-gestational-age (SGA) fetuses are not all the result of pathology and can develop as a result of normal biologic conditions; however, various factors, such as maternal disorders, infectious disease, teratogen exposure, multiple gestation, genetic disorders, and placental conditions, may contribute to the development of FGR (Gaccioli and Lager 2016).Growth-restricted fetuses haveincreased risks ofstillbirth, neonatal mortality, and morbidity(Jaddoe et al. 2014; Burton et al. 2016).Furthermore, growth-restricted fetuses are more likely to suffer from diseases, such as obesity, type 2 diabetes mellitus, coronary artery disease, and stroke, during adulthood under the long-term influence of restricted growth and altered metabolism in the fetal, neonatal, and adult periods(Barker 2006; Sailasree et al. 2017).Meanwhile, epigenetic regulation is critical for appropriate gene expression for normal functionsduring fetusdevelopment(Gicquel et al. 2008). A variety of epigenetic factors are known to interact with environmental fluctuations and regulate gene expression comprehensively(Perez and Lehner 2019). The problem with epigenetic regulation is fatal to fetus development, and it seriously affects quality of life throughout a person’s lifetime (Waterland 2009). In this study, we tried to discoverepigenetic and transcriptomic alterationsassociated with FGR development by analyzing paired DNA methylome and the transcriptome of placentae and cord bloodfrom FGR samples and normal controls.

## Results

### Differentially methylated regions were identified in placentae and cord blood of FGR samples

**Figure 1.**
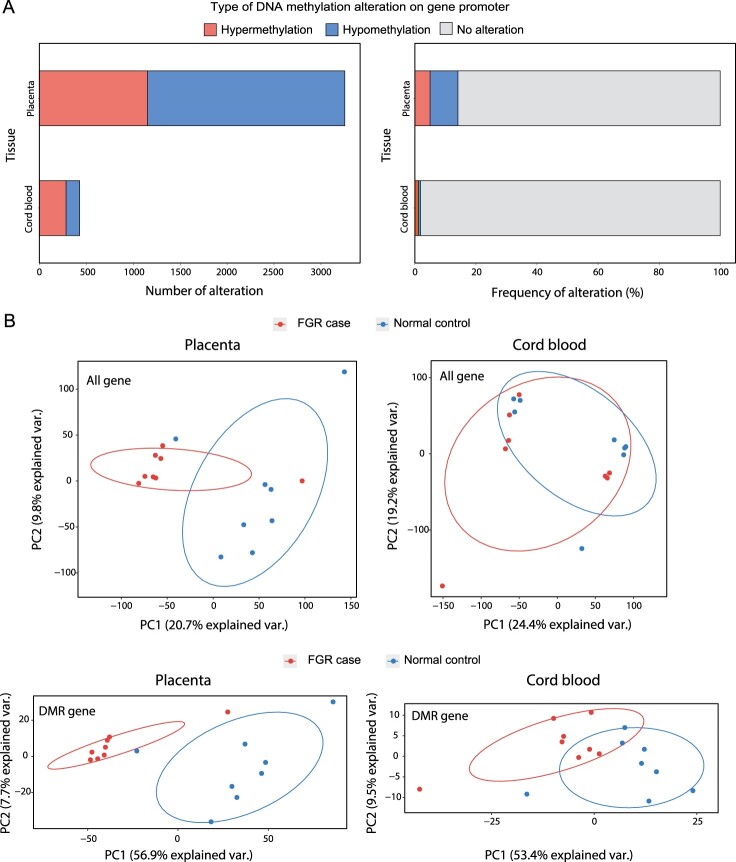
Epigenetic alteration in placentae and cord blood of FGR samples. (A) The number (left) and frequency (right) of hypermethylated and hypomethylated genes that had differentially methylated regions on the relevant promoters. (B) Principle component analysis (PCA) with DNA methylation variables were performed for all gene promotersfound in placentae (top left) and cord blood (top right). PCA was also performed for genes with differentially methylated region (DMRs) on their promoters found in placentae (bottom left) and cord blood (bottom left).

To unravel the epigenetic alterations underlying the FGR samples, we profiled DMRs for 23,049 gene promotersbetween the FGR and control samples (see Materials and Methods). We analyzed placentae and cord bloodfrom which 3,255 (14.12% of all genes) and 430 (1.87% of all genes) genes were identified as significantly hyper- or hypo-methylated, respectively (Figure 1A). The methylation status of these DMRs were tested to determine whether it had potential for classifying FGR samples from normal controls. Principle component analysis (PCA) revealed that the methylation signals of significantly methylated promoters marginally clustered FGR samples, although the methylation signals of all promoters could not classify the two groups in both placentae and cord blood (Figure 1B). Interestingly, we also discovered significantly methylated DMRs of gene bodiesand performed PCA, but the results were not grouped better than thosebased on the promoters (Supplementary Figure 1).

### A set of DNA methylation alterations wasassociated withdifferentially expressed genes in FGR samples

**Figure 2.**
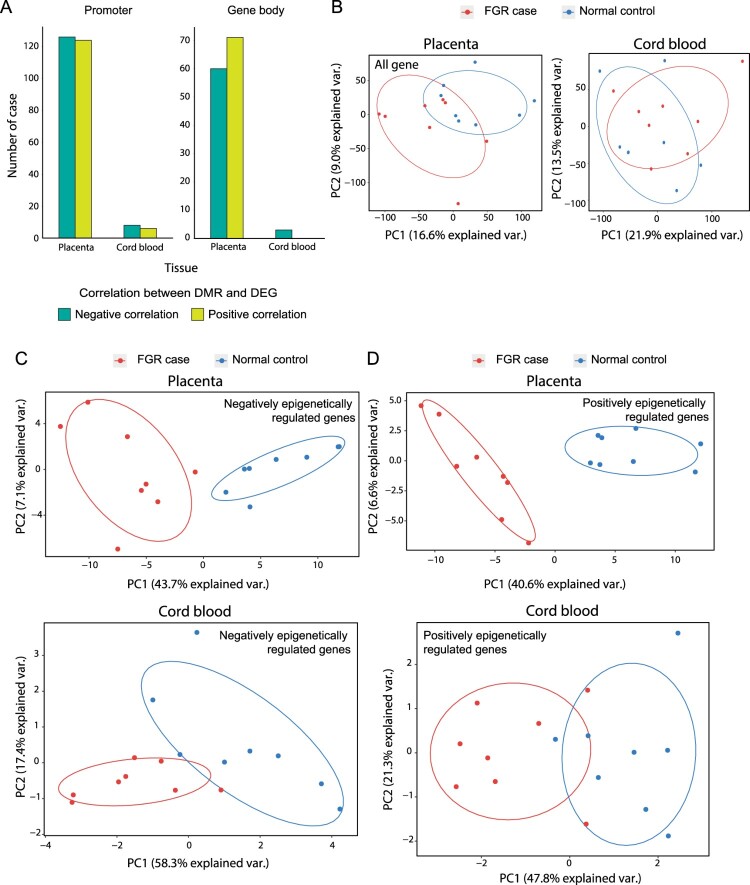
Transcriptional perturbations associated with epigenetic dysregulation in placentae and cord blood of FGR samples. (A) The ratio of differentially expressed genes (DEGs) from genes with differentially methylated region (DMRs) on their relevant promoters and gene bodies. The ratio was calculated based on negative and positive correlations between DEGs and DMRs. (B) Principle component analysis (PCA) with expression variables were performed for all genes found in placentae (left) and cord blood (right).PCA was also performed forDEGswith DMRs that correlated negatively (C) or positively (D) with each other.

DNA methylation in promotersis principally known to have a negative correlation with gene expression. Therefore, we discovered overexpressed and underexpressed genes in FGR samples accordinglyusing the hypermethylated and hypomethylated promoters, respectively. In the placentae and cord blood, respectively, 250 and 14 differentially expressed genes (DEGs) were identified with DMRs, which had 126 (50.40%) and 8 (57.14%) DEGs that could be explained by DMRs on the relevant promoters that showed a negative correlation with each other (Supplementary Table 1). In contrast to the promoter, DNA methylation on gene bodies remains to be elucidated for its regulatory functionality in transcriptional regulation(Jjingo et al. 2012). We analyzed DEGs with the status of gene body methylationsin FGR samples and found that fewerDEGs were negatively associated with methylation alteration than those of promoter methylations in placentae, although there were only negative correlations in cord bloodfor asmall number of cases (Figure 2A).PCA was also performed for the expression of all genes, and they were not clustered between FGR samples and normal controls (Figure 2B).However, the expression of DEGs with DMRs on their promoters specifically grouped FGR samples in the PCA result when the alteration of methylations was negatively correlated with the direction of DEGs (Figure 2C).On the contrary, the PCA result for the expression of DEGs having positively correlated DMRs showed worse grouping in cord bloodbut successful grouping in placentae (Figure 2D). We profiled eigen values for the genes in PCA by eigen decomposition analysis to calculate how much each gene contributed to the grouping of FGR samples. The genes with an eigen value over 0 were considered as contributing to the grouping in PCA. Then, we performed the biological pathway enrichment test for the two cases that were positively and negatively epigenetically regulated genes.This analysis could not be performed for the samples from cord blood because they had too few genes that could be profiled. As a result, we found that only cases having a negative correlation between DMRs and DEGs showed significant enrichment in the pathways related to metabolism and developmentthat were known to be associated with FGR occurrence (Supplementary Figure 2). These results imply that DMRs ongene promoters would have biological relevance in FGR occurrence by perturbing gene expressions negatively.

### Transcriptionally altered genes by DNA methylation were enriched in specific pathways relevant to FGR

**Figure 3.**
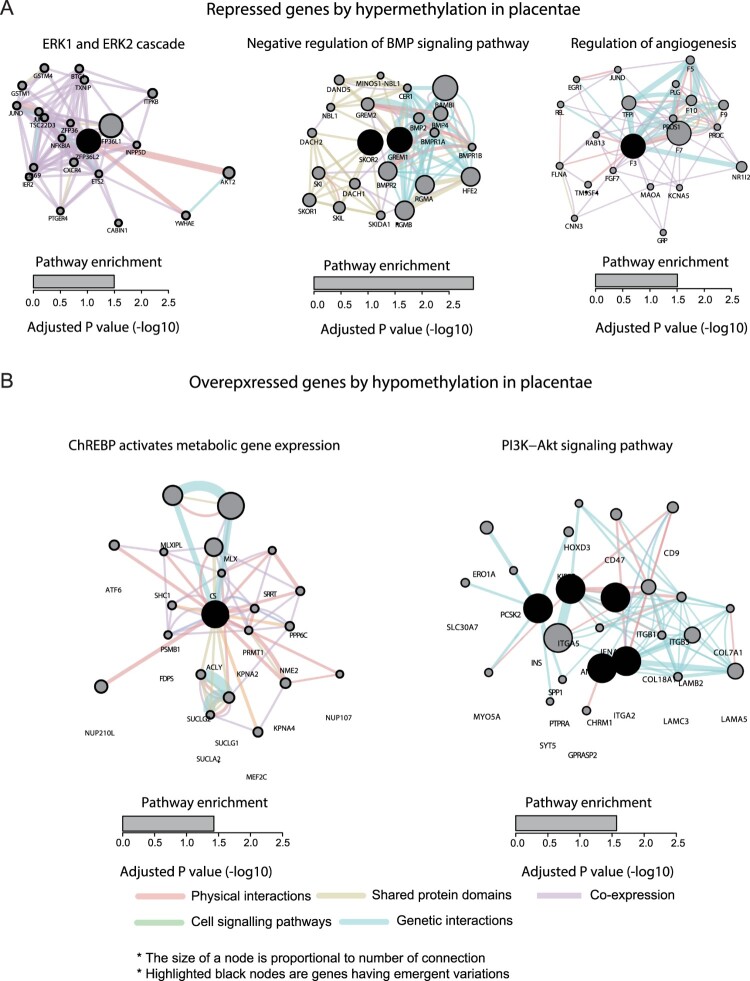
Systematic interaction of epigenetically dysregulated genes in FGR samples. The underexpressed genes with hypermethylation (A) and the overexpressed with hypomethylation in placentae from FGR samples were analyzed for pathway enrichment using the enrichR tool. Detailed interacting landscapes of thesignaling pathway enriched by the identified genes were described by the Cytoscape tool with the Genemania plugin. Physical interactions, shared protein domains, cell-signaling pathways, and genetic interactions were colored by red, yellow, green, and blue, respectively. The size of each gene node was decided accordingly by their number of interactions between other genes in the relevant pathway. The black node indicates the identified genes in FGR samples.

We analyzed how the genes that we discovered were involved in biological pathways relevant to FGR occurrence (see Materials and Methods).Genes repressed by DNA hypermethylation in placentae were enriched in the pathways that were implicated with FGR, which werethe ERK1 and ERK2 cascade(Ozmen et al. 2011), the negative regulation of BMP signaling pathway(Reid et al. 2012), and the regulation of angiogenesis(Ahmed and Perkins 2000)(Figure 3A). We also discovered thatgenes overexpressed by DNA hypomethylation in placentae were enriched in the pathways related to a set of metabolism pathways, such as the ChREBPactivation of metabolic gene expression and thePI3K-Akt signaling pathway(Figure 3B). These metabolic pathways are known to have functional implications with FGR occurrence(Zinkhan et al. 2018; Xing et al. 2019). In the case of genes underexpressed by DNA hypermethylation in cord blood, pathways related to lipid metabolism, such as the glycerolipid biosynthetic process, glycerophospholipid metabolic process, phospholipid biosynthetic process, and the regulation of the lipid metabolic process, were found to be affected. There were no significantly enriched pathways that were related to the genes overexpressed by DNA hypomethylation in cord blood.We also could not discover concordant pathways of differentially epigenetically regulated genes between placentae and cord blood.Although there was a discrepancy in the pathway enrichment between placentae and cord blood, our results indicate that the differentially epigenetically regulated genes in FGR samples are associated with a set of metabolism pathways,including the known pathways related to FGR occurrence.

## Discussion

The fetus in a placenta interacts with a variety of environments via cord blood. Several environmental signals could cause epigenetic variations that might be critical to the development and growth of fetus. In this study, we discovered a set of DMRs on the gene promoters of placentae and cord blood in FGR samples. Additionally, relevant expression perturbations were identified by changes in DNA methylation. We found out that FGR and normal control samples were classified in the PCA profiles when their gene expressions were measured for the DEGs having DMRs on their promoters. The gene expressions of all genes of the FGR samples were not classified from control samples, indicating that the genes regulated by DNA methylation are involved in FGR development. However, only a subset of genes wassignificantly differentially expressed among those having DMRs, implying that the regulation of promoter methylation is greatlycomplicated. We also discovered the pathways for differentially epigenetically regulated genes in FGR samples. A setof metabolism pathways and specific signaling pathways were identified as enriched for FGR occurrence, although they were not completely coincident between placentae and cord blood.These pathway enrichment results imply that the perturbation of DNA methylation could cause FGR development through thealteration of gene expressions.

Meanwhile, genetic imprinting is an epigenetic process including DNA methylation that causes a gene to be expressed in a specific allele (Wood and Oakey 2006). A set of imprinted genes are known to be important for normal development and placenta growth (Isles and Holland 2005; Plasschaert and Bartolomei 2014). Therefore, the inappropriate imprinting of developmental genes that perturb their expressions would be associated with FGR occurrence (Diplas et al. 2009). In this rationale, we profiled whether thegenes perturbed by DNA methylation in FGR samples were matched to known imprinting genes. The curated lists of the human imprintome(Skaar et al. 2012)are composed of 213 genes, including 107 gold-standard genes and 106 candidate genes. After matching the imprinting genes with the differentially epigenetically regulated genes in our FGR samples, three genes were identified from the placentae, which were INS, MEG3, and ZFP36L2. INS, a gene coding insulin, is crucial in glucose metabolism and is known to be involved in the regulation of fetus development (McMinn et al. 2006). MEG3 was found to be repressed by DNA hypermethylation on its promoter in our FGR placenta samples, which is consistent with the results of a previous study that described theunderexpression of MEG3 in FGR placentae(McMinn et al. 2006). ZFP36L2 is a member of the ZFP36 family and is a transcription factor regulating the response for the growth factor. It was reported that ZFP36L2 is associated with the regulation of the early developmental stage in a mouse model(Hacker et al. 2010). In our study, ZFP36L2 was down regulated by DNA hypermethylation in FGR placenta samples. These three genes are proposed as candidates for the perturbed imprinting genes related to FGR development. However, we did not have sequencing data to unravel allele-specific expression, so we could not explain which exact allele from the parents is involved in imprinting regulation.

## Materials and Methods

### IRB

This study was approved by the Institutional Review Board of Inje University Busan Paik Hospital (approval number: 18-0069)

### Samplecollection

This was case-control studythat included 8 patients with FGR and 8 normal controls delivered between 24 and 42 weeks of gestation. The FGR group included women who delivered low-birth-weight infants below the 10th percentile. The control group was comprised of singleton pregnancies without FGR.All clinical samples were collected at the Department of Obstetrics and Gynecology, Inje University, Korea. Blood samples were collected from studied women before delivery. We evaluated maternal clinical characteristics, such as age, gestational age at delivery, parity, pre-pregnancy body mass index (BMI; kg/m2), mode of delivery, frequency of preterm delivery, gestational diabetes mellitus, preeclampsia, and fetal birth weight. Preterm delivery was defined as delivery that occurred between 24 + 0 and 36 + 7 weeks of gestation. Preeclampsia was defined as the presence of hypertension with proteinuria (urine protein content >300 mg/24 h or protein/creatinine ratio ≥ 0.3) or a systemic symptom. We also evaluated the neonatal outcomes, including birth weight, neonatal death, neonatal intensive care unit (NICU) admission, Apgar score at 5 minutesafter birth, mechanical ventilation, respiratory distress syndrome (RDS), intraventricular hemorrhage (IVH), and necrotizing enterocolitis (NEC), of the FGR and control groups. Neonatal death was defined as the death of a live-born neonate within 28 days of delivery. RDS was diagnosed by compatible chest X-ray findings and arterial blood gas results. IVH was detected by bleeding inside or around the ventricles in the brain via postnatal cranial ultrasonography, whereas NEC was diagnosed via clinical and abdominal X-ray findings.

### Sample characteristics

The maternal and neonatal clinical characteristics of the study population are presented in Table 1. There were no significant differences in maternal age, gestational age at delivery, parity, BMI, mode of delivery, preterm delivery, or gestational diabetes between the FGR and control groups. Women in the FGR group had higher rates of preeclampsia than those in the control group (75.0% vs. 12.5%, p=.0147). Birth weights were significantly lower in the FGR group than in the control group (1568.75 ± 795.60 vs. 2872.50 ± 702.86gm, p=.0037). There was no neonatal death in this studied group. The FGR neonates had higher rates of neonatal complications than the control group (87.5% vs. 37.5%, p=.0455). For other neonatal complications, there were no significant differences between the two groups.

**Table 1.** Maternal and neonatal clinical characteristics.

Characteristics	FGR group (*n* = 8)	Control group (*n *= 8)	*p*-value
Maternal characteristics			
Maternal age (years)	34.50±4.81	35.62±3.77	0.6110
GA at delivery(weeks)	33.68±4.60	36.62±2.64	0.1399
Nulliparity	5(62.5%)	3(37.5%)	0.3329
Pre-pregnancy BMI (kg/m²)	21.94±3.53	24.23±4.22	0.2578
Cesarean delivery	8(100%)	8(100%)	1.0000
Preterm delivery	5(62.5%)	3(37.5%)	0.3329
GDM	1(12.5%)	0	0.3173
Preeclampsia, mild or severe	6(75.0%)	1(12.5%)	0.0147
Neonatal outcomes			
Birth weight(grams)	1568.75±795.60	2872.50±702.86	0.0037
Neonatal death	0	0	1.0000
NICU admission	7(87.5%)	3(37.5%)	0.0455
5min Apgar score <7	3(37.5%)	0	0.0628
Mechanical ventilation	4(50.0%)	3(37.5%)	0.6256
Respiratory distress syndrome	4(50.0%)	3(37.5%)	0.6256
Intraventricular hemorrhage	0	0	1.0000
Necrotizing enterocolitis	1(12.5%)	0	0.3173

### Statistics

Data were analyzed using MedCalc version 11.0 software (Frank Schoonjans, University of Gent, Belgium). Continuous variables were calculated using Student’s t-tests and Mann–Whitney U tests. Categorical variables were compared using chi-squared or Fisher’s exact tests.

### Identification of differentially methylated genes

DNAs extracted fromplacentae and cord blood were analyzed by using the Infinium HumanMethylationEPICBeadChip (Illumina,San Diego, CA, USA).A set of methylation signals on the common promoter and gene body were merged as a maximum value to determine the differentially methylated region (DMR), and the difference in methylation between 8 FGR and 8 control samples for a DMR was calculated by a difference of the mean value of each case and the control group. The Wilcoxon test was performed to calculate the statistical significance of the difference. The states of hypermethylation and hypomethylation were defined when the difference between the mean methylation value of the cases and controls was greater than +0.05 and less than -0.05, respectively.

### Identification of differentially expressed genes

For transcriptome analysis, mRNAs were extracted fromplacentae and cord blood and were analyzed by using theGeneChip Human Gene 2.0 ST Array (Affymetrix) as described by the manufacturer. Differentially expressed genes (DEGs) were analyzed using transcriptome profiles of 8 FGR samples and 8 controls who also had DNA methylome data. The DEGs that showed a significance in their expression difference were determined to be under a p value of 0.05 based on DEG analysis by Student’s t-tests. We also defined overexpressed and underexpressed genes when the -log2 transformed the fold change between mean expression value of cases, and controls was more than 1 and less than -1, respectively.

### Principal component analysis for expression and methylation variables

Principal component analysis (PCA) was used to confirm whether the variables of gene expression or methylation of FGR samples and controls were adequate for classifying each group. All expression and methylation variables were normalized using the Python StandardScaler statistics library.

### Pathway enrichment analysis

We performed pathway enrichment analysis using the EnrichR tools (Kuleshov et al. 2016), which contain a large collection of diverse gene set libraries available for pathway enrichment analysis. We used four pathway databases from EnrichR: KEGG,Reactome, Panther, and Gene Ontology Biological Process.

## Supplementary Material

Supplemental Material

## References

[CIT0001] Ahmed A, Perkins J. 2000. Angiogenesis and intrauterine growth restriction. Best Pract Res Clin Obstet Gynaecol. 14(6):981–998. 10.1053/beog.2000.0139 11141345

[CIT0002] American College of Obstetricians. 2013. ACOG practice bulletin no. 134: fetal growth restriction. Obstet Gynecol. 121(5):1122–1133. 23635765 10.1097/01.AOG.0000429658.85846.f9

[CIT0003] Barker DJP. 2006. Adult consequences of fetal growth restriction. Clin Obstet Gynecol. 49(2):270–283. 16721106 10.1097/00003081-200606000-00009

[CIT0004] Burton GJ, Fowden AL, Thornburg KL. 2016. Placental origins of chronic disease. Physiol Rev. 96(4):1509–1565. 27604528 10.1152/physrev.00029.2015 PMC5504455

[CIT0005] Diplas AI, Lambertini L, Lee MJ, Sperling R, Lee YL, Wetmur J, Chen J. 2009. Differential expression of imprinted genes in normal and IUGR human placentas. Epigenetics. 4(4):235–240. 19483473 10.4161/epi.9019

[CIT0006] Gaccioli F, Lager S. 2016. Placental nutrient transport and intrauterine growth restriction. Front Physiol. 7(FEB):1–8. 26909042 10.3389/fphys.2016.00040 PMC4754577

[CIT0007] Gicquel C, El-Osta A, Le Bouc Y. 2008. Epigenetic regulation and fetal programming. Best Pract Res Clin Endocrinol Metab. 22(1):1–16. 18279777 10.1016/j.beem.2007.07.009

[CIT0008] Hacker C, Valchanova R, Adams S, Munz B. 2010. ZFP36L1 is regulated by growth factors and cytokines in keratinocytes and influences their VEGF production. Growth Factors. 28(3):178–190. 20166898 10.3109/08977190903578660

[CIT0009] Isles AR, Holland AJ. 2005. Imprinted genes and mother-offspring interactions. Early Hum Dev. 81(1 SPEC. ISS):73–77. 15707717 10.1016/j.earlhumdev.2004.10.006

[CIT0010] Jaddoe VWV, De Jonge LL, Hofman A, Franco OH, Steegers EAP, Gaillard R. 2014. First trimester fetal growth restriction and cardiovascular risk factors in school age children: population based cohort study. BMJ [Internet]. 348(January):1–11. doi:10.1136/bmj.g14. PMC3901421 24458585

[CIT0011] Jjingo D, Conley AB, Yi SV, Lunyak VV, King Jordan I. 2012. On the presence and role of human gene-body DNA methylation. Oncotarget. 3(4):462–474. 22577155 10.18632/oncotarget.497 PMC3380580

[CIT0012] Kuleshov MV, Jones MR, Rouillard AD, Fernandez NF, Duan Q, Wang Z, Koplev S, Jenkins SL, Jagodnik KM, Lachmann A, et al. 2016. Enrichr: a comprehensive gene set enrichment analysis web server 2016 update. Nucleic Acids Res. 44(W1):W90–W97. 27141961 10.1093/nar/gkw377 PMC4987924

[CIT0013] McMinn J, Wei M, Schupf N, Cusmai J, Johnson EB, Smith AC, Weksberg R, Thaker HM, Tycko B. 2006. Unbalanced placental expression of imprinted genes in human intrauterine growth restriction. Placenta. 27(6–7):540–549. 16125225 10.1016/j.placenta.2005.07.004

[CIT0014] Minior VK, Divon MY. 1998. Fetal growth restriction at term myth or reality. Obstet Gynecol Surv. 92(1):57–60. 10.1016/s0029-7844(98)00118-5 9649093

[CIT0015] Ozmen A, Unek G, Kipmen-Korgun D, Korgun ET. 2011. The expression of Akt and ERK1/2 proteins decreased in dexamethasone- induced intrauterine growth restricted rat placental development. J Mol Histol. 42(3):237–249. 21512721 10.1007/s10735-011-9328-4

[CIT0016] Perez MF, Lehner B. 2019. Intergenerational and transgenerational epigenetic inheritance in animals. Nat Cell Biol [Internet]. 21(2):143–151. doi:10.1038/s41556-018-0242-9. 30602724

[CIT0017] Plasschaert RN, Bartolomei MS. 2014. Genomic imprinting in development, growth, behavior and stem cells. Dev. 141(9):1805–1813. 10.1242/dev.101428 PMC3994769 24757003

[CIT0018] Reid MV, Murray KA, Marsh ED, Golden JA, Simmons RA, Grinspan JB. 2012. Delayed myelination in an intrauterine growth retardation model is mediated by oxidative stress upregulating bone morphogenetic protein 4. J Neuropathol Exp Neurol. 71(7):640–653. 22710965 10.1097/NEN.0b013e31825cfa81 PMC3390978

[CIT0019] Sailasree SP, Srivastava S, Mishra RK. 2017. The placental gateway of maternal transgenerational epigenetic inheritance. J Genet. 96(3):465–482. 28761010 10.1007/s12041-017-0788-5

[CIT0020] Skaar DA, Li Y, Bernal AJ, Hoyo C, Murphy SK, Jirtle RL. 2012. The human imprintome: regulatory mechanisms, methods of ascertainment, and roles in disease susceptibility. ILAR J. 53(3–4):341–358. 23744971 10.1093/ilar.53.3-4.341 PMC3683658

[CIT0021] Waterland RA. 2009. Is epigenetics an important link between early life events and adult disease? Horm Res. 71(SUPPL. 1):13–16. 19153498 10.1159/000178030

[CIT0022] Wood AJ, Oakey RJ. 2006. Genomic imprinting in mammals: emerging themes and established theories. PLoS Genet. 2(11):1677–1685. 10.1371/journal.pgen.0020147 PMC1657038 17121465

[CIT0023] Xing Y, Zhang J, Wei H, Zhang H, Guan Y, Wang X, Tong X. 2019. Reduction of the PI3 K/Akt related signaling activities in skeletal muscle tissues involves insulin resistance in intrauterine growth restriction rats with catch-up growth. PLoS One. 14(5):1–16. 10.1371/journal.pone.0216665 PMC6508867 31071176

[CIT0024] Zinkhan EK, Yu B, Schlegel A. 2018. Prenatal exposure to a maternal high fat diet increases hepatic cholesterol accumulation in intrauterine growth restricted rats in part through microRNA-122 inhibition of Cyp7a1. Front Physiol. 9(MAY):1–10. 29896121 10.3389/fphys.2018.00645 PMC5987111

