# Supplementary figures and images for: DNA Methylation and gene expression patterns are widely altered in fetal growth restriction and associated with FGR development
Source: Anim Cells Syst (Seoul). 2021 May 17;25(3):128–35. doi: 10.1080/19768354.2021.1925741 (PMC8253195; doi:10.1080/19768354.2021.1925741)

Supplementary Figure 1

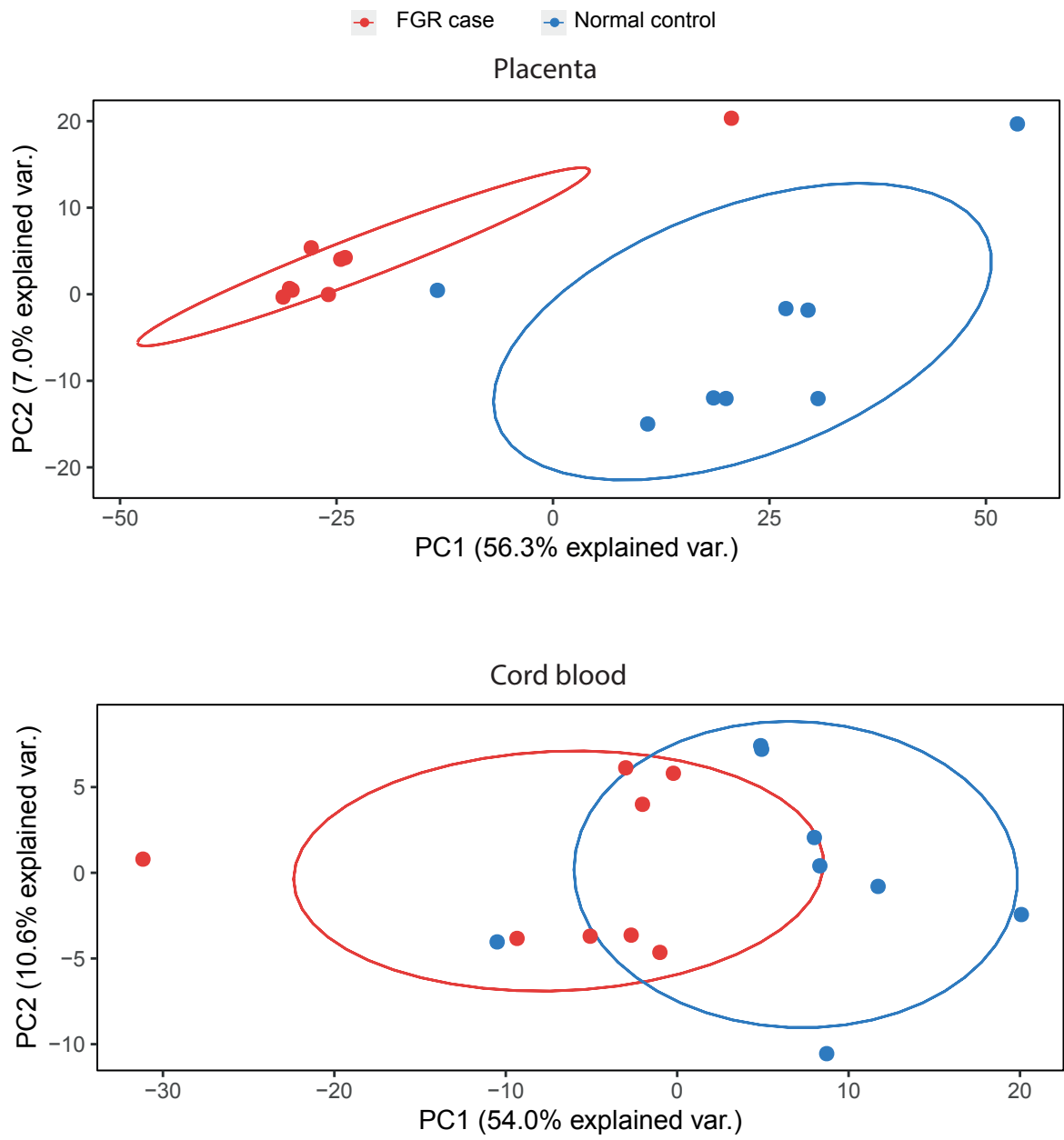

Supplementary Figure 2

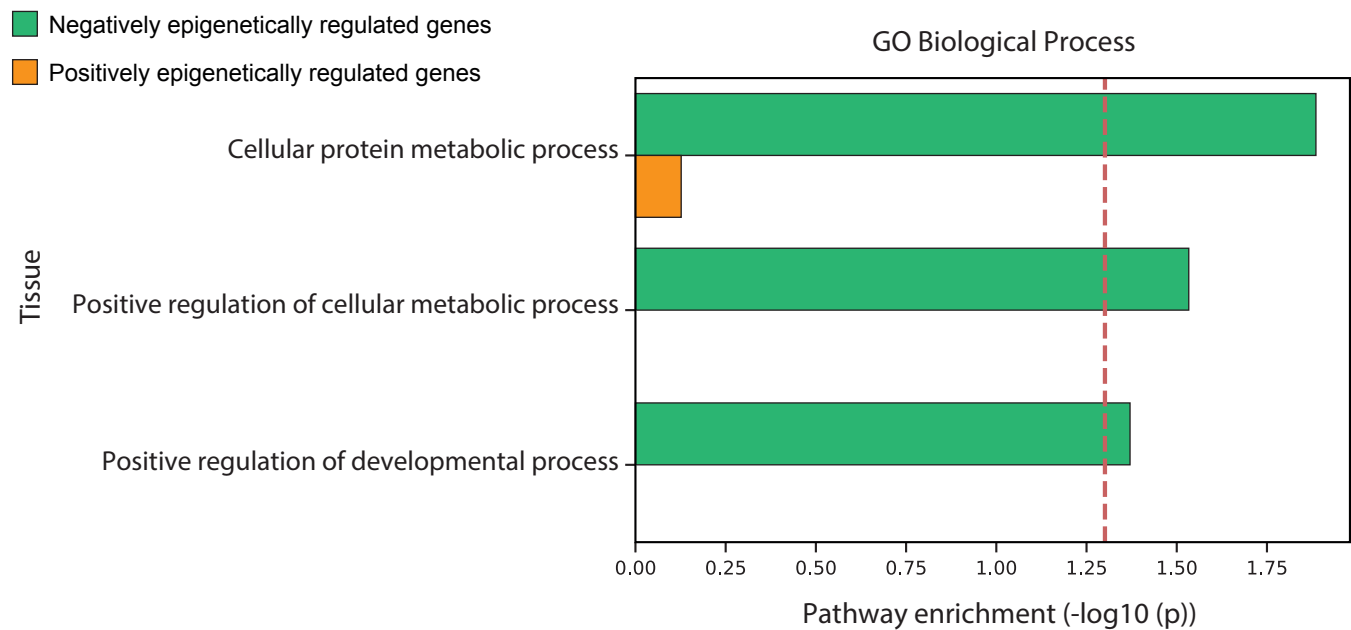

Supplement: Supplemental Material [file TACS_A_1925741_SM4659.pdf]
